# Injection Versus Ingestion: A Systematic Review of the Cost Burden of Long-Acting Injectable Antipsychotics Compared to Oral Antipsychotics

**DOI:** 10.1192/bjo.2026.11140

**Published:** 2026-06-30

**Authors:** Gaurav Uppal, Asha Dhandapani, Sathyan Soundararajan, Naima Gul, Safa Puliyakkadi

**Affiliations:** 1 Satyam Hospital, Ludhiana, India; 2 BCUHB, Wrexham, United Kingdom; 3 Leeds Community Healthcare Trust, Leeds, United Kingdom

## Abstract

**Aims:**

Due to their higher acquisition costs, long-acting injectable (LAI) antipsychotics are often seen as more expensive substitutes for oral antipsychotics (OAs). However, Schizophrenia is a chronic and relapsing- remitting illness; non-compliance to oral therapy frequently results in hospitalisation and emergency care, which are significant contributors to overall healthcare expenses. Therefore, rather than concentrating solely on pharmacies, evaluating treatment value necessitates a total cost of care (TCOC) perspective.

Aims were to determine, using economic data, whether the higher costs of LAI antipsychotics result in an overall increased healthcare cost when compared to oral antipsychotics in adults with Schizophrenia.

**Methods:**

Our data sources for the study included administrative claims databases (Medicaid and commercial), cost-effectiveness models, mirror-image (pre-post) studies, national health system cohorts, systematic reviews and meta-analyses. We conducted a structured narrative evidence synthesis from the above data.

We focused on real-world economic and pharmacoeconomic studies comparing LAI antipsychotics with OAs. The total healthcare costs and/or component costs (pharmacy, inpatient, emergency department, and outpatient) were reported in the studies. Results were analysed directionally rather than pooling them statistically, due to heterogeneity in settings,currencies, and time horizons.

**Results:**

It was noted that across US, European, and Asian studies, LAIs consistently demonstrated lower inpatient and emergency department costs but higher pharmacy costs in comparison to OA’s.

Several pre-post analyses revealed a reduction in hospitalisation costs after LAI initiation. However, few claims-based and meta-analytic studies reported no statistically significant difference in total healthcare costs between LAIs and oral therapy.

Cost-effectiveness models indicated that LAIs may be cost-effective over multi-year horizons, despite similar or slightly higher total costs, driven by improved compliance and reduced relapse rates.

The findings demonstrate a robust “cost-offset effect”, in which the higher upfront medication costs of LAIs are balanced by the downstream savings from avoided hospitalisations.

The generalisability of this pattern for stakeholders and policy/decision-makers was supported by the fact that it was consistent across healthcare systems, study designs, and LAI molecules.

**Conclusion:**

LAI antipsychotics do not significantly raise overall healthcare costs and may be economically beneficial by lowering expensive relapse-related care, though they may appear to be raising pharmacy expenditures initially. Treatment evaluations for schizophrenia should focus on the overall cost of care rather than just the cost of the medication.

